# MicroRNAs targeting *CDKN2A* gene as a potential prognostic marker in head and neck squamous cell carcinoma

**DOI:** 10.22099/mbrc.2023.48081.1853

**Published:** 2024

**Authors:** Sivakumar Gopalakrishnan, Anitha Pandi, Paramasivam Arumugam, Vijayashree Priyadharsini Jayaseelan

**Affiliations:** 1 Clinical Genetics Lab, Centre for Cellular and Molecular Research, Saveetha Dental College & Hospital, Saveetha Institute of Medical and Technical Sciences [SIMATS], Saveetha University, Chennai, India; 2 Department of Oral Pathology and Microbiology, Madha Dental College and Hospital, Kundrathur, Chennai-69; 3 Molecular Biology Lab, Centre for Cellular and Molecular Research, Saveetha Dental College & Hospital, Saveetha Institute of Medical and Technical Sciences [SIMATS], Saveetha University, Chennai, India

**Keywords:** Cancer, Gene expression, microRNA, HNSCC, Survival, Novel biomarkers

## Abstract

Epigenetic factors are known to markedly influence the functions of a gene by modification of transcripts, *via* methylation or acetylation and degradation of mRNA transcripts. The *CDKN2A* encodes cyclin-dependent kinase inhibitor 2A, a tumour suppressor protein. Genetic and epigenetic alterations in this gene have been demonstrated in several cancer types. The non-coding RNAs with a special emphasis on microRNAs have long been explored for their potential role in the epigenetic modification of gene expression. The present study aims to identify the microRNAs targeting *CDKN2A *gene transcripts and demonstrate their prognostic significance in head and neck squamous cell carcinoma (HNSCC). Computational approaches were employed to identify the microRNAs targeting *CDKN2A. *The gene and protein expression profile of *CDKN2A* was analyzed using UALCAN. A significant upregulation of *CDKN2A* was observed in the primary tumour tissues (p=<10^-12^). Interestingly, the protein expression, although found to be statistically significant (p=0.0129) did not correlate well with the gene expression profile. The microRNAs targeting *CDKN2A *were further analyzed to identify the possible reason for the decrease in protein expression. Among the 44 microRNAs targeting *CDKN2A* gene transcripts, hsa-miR-3681-3p, hsa-miR-542-5p, hsa-miR-4519 were found to be upregulated and hsa-miR-134-5p was found to be downregulated with a significant association with survival status of HNSCC patients. The hsa-miR-542-5p was found to correlate well with the survival and hence can be considered as the key microRNA associated with HNSCC. However, further validation of this microRNA is warranted to confirm its role in the process of carcinogenesis.

## INTRODUCTION

Head and neck squamous cell carcinoma (HNSCC) is a group of complex heterogeneous cancers involving the malignant transformation of the cells that line the aerodigestive tract, such as the nasal or oral cavity, pharynx, oropharynx, larynx, paranasal sinus, salivary glands and localized lymph nodes. The alterations in the genetic and epigenetic landscape are a common presentation in the case of any cancer type [1]. This can lead to genomic instability, abnormal proliferative cycles, cell death evasion, altered metabolism etc., [2]. The HNSCC is the common malignant phenotype observed in the Asian population with a high incidence rate. Oral cancer constitutes about 16.1% of all cancers affecting males and is the 4^th^ most frequently encountered cancer type among women in India (http://cancerindia.org.in/cancer-statistics/). 

Despite the availability of therapeutic modalities, lack of awareness and chronic habitual practices such as tobacco chewing and smoking have hampered the efforts to improve the survival probability of patients suffering from HNSCC [3]. In addition to the current status, the incidence rate of oral cancer in young adults and individuals developing HNSCC with no history of risk-associated habits has markedly influenced the management of this cancer type [4]. It has been reported that the 5-year survival rate of early-stage oral cancer patients is around 82%, while it drastically reduces to 27% in advanced stages, underscoring the need to develop early diagnostic tools and targeted therapy approach for prompt treatment and management (http://cancerindia.org.in/cancer-statistics/). 

The genes that are reported to be more often linked to cancer phenotypes are *TP53*, *CDKN2A*, *PIK3CA*, *NOTCH1, EGFR *and *CCND1. *These genes were found to harbour gross chromosomal abnormalities such as deep deletion, amplification, translocation and single nucleotide substitutions such as missense, nonsense, splice site, and frameshift mutations [1]. Several genes act as tumour suppressors, while other genes are major regulators of the hallmarks of cancer. In this context, *CDKN2A* has been investigated in numerous cancer types to reveal its functions in carcinogenesis. *CDKN2A* codes for the protein, cyclin-dependent kinase inhibitor 2A, which is a potent tumour suppressor protein. The *CDKN2A* are known to regulate two crucial pathways involved in the process of carcinogenesis *viz.,* the p53 and RB1 pathways. The alternative splicing process generates 2 major proteins, p16 (INK4) and p14 (ARF) that bind to MDM2, a p53 stabilizing protein [5]. In addition to the genetic modifications, the epigenetic mechanisms, such as methylation [6], histone acetylation [7], and interaction of non-coding RNAs with the gene transcripts lead to severe dysregulation of molecular pathways that control the normal cell processes [8]. The most common and widely explored epigenetic component is the microRNA, which is a single-stranded, small oligonucleotide sequence with the ability to bind to and bring about translational inhibition of target mRNA [9]. The differential expression of microRNAs targeting a specific gene in HNSCC is a potential candidate as prognostic, diagnostic or therapeutic leads. The upregulated microRNAs are OncomiRs, which can induce translation by degrading the target mRNA transcripts. Certain other miRNAs can exhibit a tumour-suppressive effect by targeting the candidate Oncogene transcripts, eventually leading to translational inhibition [10]. Researchers have reported the role of miRNAs targeting key genes of the cancer pathways, such as the TP53 pathway, EGFR pathway, Mesenchymal-epithelial transition factor (c-Met) pathway, Insulin growth factor 1 receptor pathway, hypoxia and angiogenesis pathway etc., [11]. 

A recent study reported that serum from HNSCC patients induced differential expression of 16 miRNAs in exposed cells when compared to serum from normal healthy individuals. Such cell-free assays can dramatically improve therapy as well as the prognosis of patients [12]. The microRNA nano-based therapies have also been investigated to improve the therapeutic paradigms for HNSCC treatment [13]. Emerging technologies have aided in the development of Peptide nucleic acid-based OncomiR inhibitors, that can be used to suppress the expression of OncomiRs thereby leading to diminished tumour growth [14]. Also, it is feasible to efficiently detect the pathological and clinical aspects of the alterations, such as proliferation, the degree of tumour heterogeneity, and the invasiveness of cancer cells, by identifying the miRNA signature for particular types of neoplastic tumours [15].

## MATERIALS AND METHODS

The microRNAs targeting *CDKN2A* were identified using miRDB, the MicroRNA Target Prediction Database (http://mirdb.org). The miRDB is an exclusive online resource for target prediction and functional annotation [16-17]. The gene name was provided as a query and the resulting microRNA list was populated by the algorithm, which was used for further analysis. The expression and survival profile of the gene and the microRNAs were analyzed using the UALCAN database. The HNSCC source data for gene expression and survival analysis were from the TCGA (The Cancer Genome Atlas) database. The web resource is an excellent tool for analysing OMICS data. A descriptive PERL module was used to calculate the interquartile ranges after filtering the outliers. The significant differences in the expression levels between the normal (n=44) and primary tumour (n=520) were estimated through Welch’s T-test. The survival analysis is an important criterion for prognostic validation of the microRNA of interest. Both univariate and multivariate analyses were used to evaluate the overall survival of patients based on their expression profiles. The samples with expression values > to the third quartile were designated as the high expression group and samples with gene expression values < third quartile were designated as low/medium expression groups. The R packages “survival” and “survminer” were used to generate the survival plots. The log-rank tests were used to determine the P values, indicating the level of significance [18]. The gene and protein expression data along with the survival rate of the HNSCC patients were analyzed with respect to the expression of their cognate microRNAs.

## RESULTS

**Figure 1:**
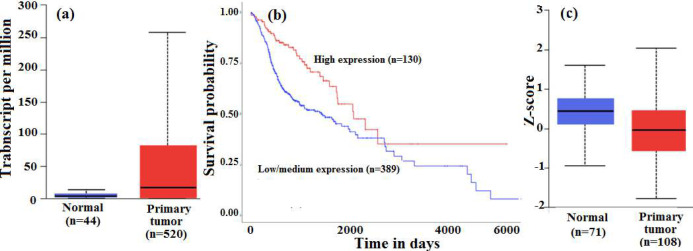
**(a)** Box Whisker plot demonstrating the expression of the *CDKN2A* gene expression in the primary tumour and normal tissues. A statistically significant difference was observed between the two groups with a p<10^-12^. There was a significant upregulation of mRNA transcripts in the HNSCC tumour group; **(b)** Kaplan Meier survival analysis demonstrating the survival of HNSCC patients with respect to the varied expression profile of *CDKN2A*. The patients presenting with low/medium level expression of *CDKN2A* were found to present with poor prognosis (p=0.00038); **(c)** Box-Whisker plot demonstrating the protein expression of CDKN2A in the primary tumour of HNSCC when compared to the normal tissues

The gene and protein expression profile of the *CDKN2A* gene demonstrated a significant increase in gene expression (p=<10^-12^) (Fig. 1a) and decreased protein expression (p=0.0129) (Fig. 1c). The contradicting presentation of the *CDKN2A *gene and protein expression demanded further investigations on the influence of epigenetic components, especially the microRNAs. Interestingly, the Kaplan-Meier survival plot also supported the fact that patients presenting with low/medium level expression of *CDKN2A* exhibited poor prognosis when compared to the group exhibiting high levels of expression (p=0.00038) (Fig. 1b). 

**Figure 2:**
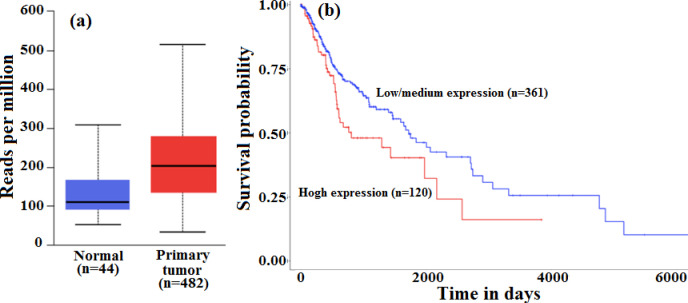
**(a)** Box Whisker plot demonstrating the expression of microRNA, hsa-miR-542, in HNSCC patients. Significant upregulation was observed in the primary HNSCC tumour group when compared to the normal tissues (p=5.318x10^-8^). **(b)** The Kaplan-Meier survival plot shows a significant difference in survival rate among the low/medium expression group and high expression group (p=0.02).

The possible reason for the decrease in the protein levels (p=0.0129) could be attributed to microRNAs targeting the *CDKN2A* gene. Around 44 microRNAs were found to target the *CDKN2A* gene, of which only 23 were found to include expression data. The other microRNAs were excluded due to lack of sufficient data for analysis. Among the 23 microRNAs, seven were found to be downregulated and 15 were found to be upregulated, with one showing insignificant expression. The microRNAs, hsa-miR-3681-3p, hsa-miR-542 and hsa-miR-4519 were found to exhibit a significant upregulation accompanied by a statistically significant survival rate in HNSCC patients. However, hsa-miR-542, returned a positive correlation between the expression profile (Fig. 2a) and survival rate, wherein, patients who presented with relatively high levels of hsa-miR-542 (p=5.318×10^-8^) exhibited poor survival rate (p=0.02) (Fig. 2b). 

## DISCUSSION

Several tumour suppressor genes such as *MTAP, CDKN2A* and *B*, mapped to the locus 9p21, were shown to present with homozygous or heterozygous deletions accompanied by poor prognosis in multiple forms of cancer [19]. The present study demonstrates the upregulation of *CDKN2A *in HNSCC patients. The expression of this gene is found to be greatly regulated by epigenetic mechanisms namely, promoter methylation, histone modification and degradation by non-coding RNAs. Hypermethylation of *CDKN2A *of promoter and loss of p16 were associated with shorter survival of CRC [20]. In contrast to these reports, increased expression of *CDKN2A* was also documented in multiple cancers. The expression profile correlated with tumour mutation burden, microsatellite instability and infiltration of lymphocytes [21].

Computational approaches have been used to explore the gene families [22-23], gene network interactions [24] and epigenetic targets [25-26] that modulate the expression of key genes involved in the process of carcinogenesis. The differentially expressed genes (DEG) associated with cancer and their target microRNAs have been analyzed to understand the role of these epigenetic marks at different stages of cancer starting from initiation to invasion. Most often, microRNAs are curated based on their differential expression profile by comparing samples from disease and healthy states. The investigations on the exosomal or circulating microRNAs targeting candidate genes involved in the process of carcinogenesis could be an alternative methodology to curate a hub of microRNAs to develop it as a unique panel for a specific dysregulated gene associated with the cancer type [27]. In view of this fact, the present study was conducted to investigate the microRNA targeting *CDKN2A* gene, which revealed one key microRNA *viz., *hsa-miR-542 that can be further developed as a prognostic, diagnostic or therapeutic lead for HNSCC. 

Several studies have implicated the tumour suppressive role of miR-542-3p in different types of cancer and that a significant downregulation was observed in most cancer types. A study conducted by Wu and team demonstrated the expression of hsa-miR-542-3p in oral squamous cell carcinoma (OSCC) patients in relation to the expression of survivin. The role of inhibitors and mimics on the expression of hsa-miR-542-3p was also investigated. The findings of the study reported decreased expression of hsa-miR-542-3p and increased expression of survivin in OSCC patients [28]. A similar study was performed by Patel and Rawal to elucidate the role of microRNA and cytokines in the modulation of the CSC (Cancer Stem Cells) signalling pathway. The study documented the increased expression of *CD44v3, CD44v6, Bmi1* and *Nanog,* with concomitant downregulation of *PTEN* and *ATM* in OSCC patients. The team also reported the decreased expression of three microRNAs *viz., *542-3p, 34a and 9. Interestingly, miR542-3p and miR34a were found to target the CD44v6-Nanog-PTEN axis, thereby regulating the CSC properties. This study provides substantial evidence about the involvement of miR542-3p in conferring stemness to the cancer cells [29]. 

Whilst numerous studies reported downregulation of miRNA-542, a few studies have reported the upregulation of miRNA-542. The miRNA 542-5p was found to be upregulated in osteosarcoma and was found to promote tumorigenesis by targeting HUWE1 [30]. A recent clinical study revealed circulating microRNAs that are differentially expressed in colorectal cancer (CRC). The microRNAs, miR-542-5p, miR-28-3p, let-7e-5p, and miR-106a-5p were identified using TaqMan Low-Density Array. The expression profile was further confirmed using qRT-PCR followed by validation using GEO Omnibus. These study findings showed that the four microRNAs identified can be used as a predictive tool for the detection of CRC [31]. 

The escalating number of HNSCC cases in the Indian population demands a promising diagnostic tool for early diagnosis. The computational approaches have aided in delineating the molecular biomarkers specific to a cancer type. The present study hypothesized that the miR-542 could be a potential candidate targeting *CDKN2A. *However, the study was bound by certain limitations,* viz.,* (a) the study design being an *in silico *approach, has to be further validated experimentally to confirm the association of miRNA targeting *CDKN2A* in association with HNSCC, (b) although the microRNA targeting *CDKN2A* was found to be miR-542-5p, the gene expression profiling database provided information on miR-542, with no specific distinction between 3p and 5p types, (c) there are other epigenetic marks such as methylation, histone modification which can interfere with the gene expression, eventually contributing to a dysregulated pathway or process. Considering both the advantages and limitations of the study design, the microRNA identified in this study should be tested further for its functional role among different cancer stages, ethnic groups, individuals with or without habits and other factors, to gain a better insight into their association with HNSCC. 

### Acknowledgement:

The authors are grateful to all the consorts and groups involved in the compilation of data from patients for public use. Our sincere thanks also go to all the patients who have indirectly contributed to the scientific community by providing consent for sharing their data for research use.

### Conflict of Interest:

The authors have no conflicts of interest relevant to this article.

### Authors’ contribution:

SG: Data Collection and Writing manuscript; AP: Conducting in silico analysis and preparation of manuscript draft; PA: Verifying the data and analysis; VPJ: Conceptualization and interpretation of results. All authors reviewed the results and approved the final version of the manuscript.
